# Avoidant/Restrictive Food Intake Disorder: A Longitudinal Study of Malnutrition and Psychopathological Risk Factors From 2 to 11 Years of Age

**DOI:** 10.3389/fpsyg.2018.01608

**Published:** 2018-08-31

**Authors:** Loredana Lucarelli, Cristina Sechi, Silvia Cimino, Irene Chatoor

**Affiliations:** ^1^Department of Pedagogy, Psychology, Philosophy, University of Cagliari, Cagliari, Italy; ^2^Department of Dynamic and Clinical Psychology, Sapienza University of Rome, Rome, Italy; ^3^Department of Psychiatry and Behavioral Sciences, The George Washington University, Washington, DC, United States

**Keywords:** avoidant/restrictive food intake disorder, infantile anorexia, malnutrition, psychopathological risk factors, longitudinal outcome

## Abstract

**Aim:** To evaluate different types and degrees of malnutrition over time in a sample of children diagnosed with Infantile Anorexia (IA), based on the DC:0-3R criteria, and recently defined by DSM-5 as the first subtype of Avoidant/Restrictive Food Intake Disorder (ARFID), and to investigate the relationship between children’s severity of malnutrition and emotional/behavioral development, and mothers’ long-term psychopathological symptoms.

**Methods:** A total of 113 children (58 boys, 55 girls), originally diagnosed with IA, and their mothers, were evaluated at four assessment points at the children’s mean age of 2, 5, 7, and 11 years. Several measures were used to assess the children’s growth and level of malnutrition, mothers’ psychopathological symptoms and eating attitudes, as well as their children’s emotional/behavioral functioning.

**Results:** A steady improvement in the severity of malnutrition over time emerged, but 73% of children still had ongoing mild to moderate to severe malnutrition at 11 years of age. Moreover, the children showed increasing internalizing and externalizing emotional/behavioral problems, and their mothers’ psychopathological symptoms and eating problems worsened as well over time. At 11 years of age, the girls’ emotional/behavioral problems and their mothers’ psychopathology and disturbed eating attitudes were more severe than that of the boys and their mothers. Finally, during the last assessment, significant associations between the mothers’ psychopathology and disturbed eating attitudes, the severity of the children’s malnutrition, and their emotional/behavioral problems emerged.

**Discussion:** Our longitudinal study points out that the developmental course of children, originally diagnosed with IA and who received limited psychosocial treatment, is characterized by an enduring risk of malnutrition and increasing psychopathological symptoms in both, the children and their mothers, up to the sensitive period of pre-puberty.

## Introduction

The study we are going to present in this paper assessed different types and degrees of malnutrition over time in a cohort of children, initially diagnosed with Infantile Anorexia (IA), and investigated the relationship between the children’s severity of malnutrition, emotional/behavioral development and their mothers’ long-term psychopathological symptoms. The diagnosis of IA was based on the DC:0-3R criteria, and recently defined by DSM-5 as the first subtype of Avoidant/Restrictive Food Intake Disorder (ARFID). Most studies of IA have been cross-sectional and have explored different types and degrees of malnutrition, infant temperament, maternal psychopathology, and mother–child interactions during feeding. However, the long-term outcome of IA is still poorly understood.

In the *Diagnostic and Statistical Manual of Mental Disorders* (DSM-5, American Psychiatric Association [APA], 2013), all feeding and eating disorders have been moved into a single Feeding and Eating Disorders section, which is consistent with the recognition of the continuity of psychopathology from early childhood to adulthood (Bryant-Waugh and Kreipe, 2012; Bryant-Waugh, 2013; Kenney and Walsh, 2013). In this section, DSM-5 provides criteria for ARFID, which replaced and extended the feeding disorder of infancy or early childhood diagnosis described in DSM-IV (American Psychiatric Association [APA], 1994).

It was well recognized that the criteria for infancy in DSM-IV were too narrow and vague and did not differentiate between various subtypes of feeding disorders, resulting in low clinical utility. For these reasons, alternative classifications have been used by clinicians to increase the specificity of the DSM-IV diagnosis (Chatoor, 2002; Chatoor and Ammaniti, 2007; Bryant-Waugh et al., 2010; Bryant-Waugh and Kreipe, 2012; Bryant-Waugh, 2013; Lucarelli et al., 2013; Bryant-Waugh and Watkins, 2015), most notably the “Feeding Behavior Disorder” section in the *Diagnostic Classification of Mental Health and Developmental Disorders of Infancy and Early Childhood-Revised* (DC:0-3R, Zero To Three, 2005), which defined six different diagnostic subtypes of feeding disorders, including IA. IA is characterized by food refusal and significant growth deficiency, the young child does not communicate hunger, lacks interest in food, but shows strong interest in exploration and interaction with the caregiver. The ARFID diagnosis in DSM-5 describes three feeding disorder subtypes from DC:0-3R; particularly, IA corresponds to the first ARFID subtype “apparent lack of interest in eating or food.”

Cross-sectional studies of IA have explored the following: malnutrition, infant temperament, maternal psychopathology, and mother–child interactions during feeding. These studies demonstrated that children with IA exhibit different degrees of acute and/or chronic malnutrition (Chatoor et al., 1998, 2000; Ammaniti et al., 2004, 2010; Lucarelli et al., 2013). In children, malnutrition manifests as underweight and/or stunting (short stature), however, during malnutrition a weight deficit is the first abnormality noted, followed by a length or height deficit. For this reason, *acute malnutrition* describes how thin the child is for his/her height and *chronic malnutrition* describes how short the child is for the child’s age; malnutrition may occur along a continuum of mild, moderate, and severe levels of acute and/or chronic malnutrition.

Further research revealed a fussy-difficult temperament and a heightened level of physiological arousal in children with IA (Chatoor et al., 2000, 2004; Lucarelli et al., 2013) and demonstrated that mothers of these children show a psychopathological profile, mainly characterized by anxiety, depression, and dysfunctional eating attitudes (Chatoor et al., 1998, 2000; Ammaniti et al., 2004, 2010; Lucarelli et al., 2013). Moreover, the feeding interactions between children with IA and their mothers have been characterized by low dyadic reciprocity, interactional conflict, and negative affect in both the children and their mothers (Chatoor et al., 1998, 2000, 2018; Ammaniti et al., 2004, 2010; Lucarelli et al., 2013; Tambelli et al., 2014). Interestingly, the level of dyadic conflict between the mother and her child with IA can be a significant factor to differentiate IA from other feeding disorder subtypes, such as *Sensory Food Aversions* or *Feeding Disorder Associated with Insults to the Gastrointestinal Tract*, also named *Post-Traumatic Feeding Disorder* (Chatoor et al., 2001; Lucarelli et al., 2013). Finally, research in the field of IA also demonstrated associations between maternal psychopathology, difficult infant temperament, and mother–child interactional conflict during feeding, and revealed that both child and maternal characteristics are significant predictors of dyadic interactional conflict during infancy (Chatoor et al., 2000; Ammaniti et al., 2004, 2010).

The long-term outcome of IA is still poorly understood. Some prospective studies of large community-based samples from early childhood to young adulthood showed that child feeding problems [as defined by Marchi and Cohen (1990), i.e., “does not eat enough,” “is often or very often choosy about food,” and “is usually not interested in food”], undereating, eating conflicts, struggles with food, unpleasant meals in early childhood, female sex, maternal weight, and depressive symptoms are risk factors for the later development of Anorexia Nervosa, and other eating disorders such as Bulimia Nervosa, in adolescence and young adulthood (Marchi and Cohen, 1990; Kotler et al., 2001; Nicholls and Viner, 2009).

A few longitudinal studies of IA that investigated a shorter period of time, i.e., from infancy to preschool age and mid-childhood (Ammaniti et al., 2012; Chatoor, 2013), showed that even when children and their parents received treatment during infancy, risk factors related to the continuity of psychopathology emerged. In a clinical sample that was treated during infancy, two-thirds of children had developed healthy eating patterns and good growth at follow-up during mid-childhood, whereas one-third of the children continued to show various degrees of poor growth (Chatoor, 2013). Both longitudinal studies (Ammaniti et al., 2012; Chatoor, 2013) demonstrated that if the parents had difficulty following treatment recommendations, the children continued to struggle with eating, showed signs of early satiety, and became increasingly fussy about food. These children also showed anxiety, moodiness, somatic complaints, oppositional behaviors, and social problems, indicating that they are at risk not only for ongoing eating problems, but also for anxiety disorders and behavioral difficulties.

Overall, current findings point to the need for continued research to better understand the biologic, emotional-behavioral, and environmental risk factors for eating disorders covering the period from early childhood to adolescence and adulthood. While it is conceivable that individuals with this subtype of ARFID, who show reduced food intake due to a general lack of appetite or interest in eating, may go on to develop another eating disorder such as Anorexia Nervosa, no longitudinal studies are yet available (Kenney and Walsh, 2013).

### The Purpose of the Study

The present study examined a cohort of children, diagnosed with IA at 2 years of age and followed them up to 11 years of age. Following a transactional model, which recognizes the complex interplay between the individual characteristics of the mother and the child in the origin of developmental psychopathology (Sameroff, 2000), we aimed to expand longitudinal research on IA by exploring whether the children’s severity of malnutrition, maternal psychopathology, and dysfunctional eating attitudes are related to the emotional development of these children from infancy to pre-puberty. We also examined whether these risk factors take on different trajectories over time related to the age and/or sex of the children. As the DSM-5 ARFID is a new diagnosis, epidemiological data are limited, but several studies show that the adolescent years are a critical period for the onset of restrictive eating disorders. (Nicholls et al., 2011; Pinhas et al., 2011; Baker et al., 2012). Moreover, the literature indicates a significantly greater incidence of Anorexia Nervosa and associated psychopathology in females (Lewinsohn et al., 2000; Currin et al., 2005; Van Son et al., 2006; Keski-Rahkonen et al., 2007), which raises the question whether female children with IA may have a greater risk for associated psychopathology as well.

We report data from toddlers with IA and ARFID/Restrictive subtype diagnosis, and their mothers, who received some psychoeducation about this specific eating disorder at the time of the diagnosis and during subsequent assessments, but did not pursue any specific psychotherapeutic treatment for various reasons: lived or moved to another city, lived in other areas of Italy without appropriate resources, refusal by one or both parents, or precocious termination of treatment. However, the children were followed closely by the pediatricians at the hospital in Rome and in their community, and they were periodically called back for subsequent appointments at the hospital in Rome. They were examined four times: at the time of diagnosis when the children were of a mean age of 2 (Assessment 1°) and at three more assessments at 5 years (Assessment 2°), 7 years (Assessment 3°), and 11 years (Assessment 4°) of age.

Specifically, this study aimed to examine the following:

(1) Do children’s malnutrition classification (acute, chronic, and combined acute/chronic) and degree of severity (mild, moderate, and severe) change over time?
(2) Is there a difference in the emotional development related to the age and sex of the children? Specifically, are girls more vulnerable than boys, if their mothers have eating and general psychopathological symptoms?
(3) Are there associations between the psychopathological profile and eating attitudes of the mothers, the children’s emotional/behavioral problems, and the children’s severity of malnutrition? Do these associations persist over time?
(4) Do the children’s severity of malnutrition and the psychopathological profile and eating attitudes of the mothers have an impact on the children’s emotional/behavioral development?

## Materials and Methods

### Participants

**FIGURE 1:**
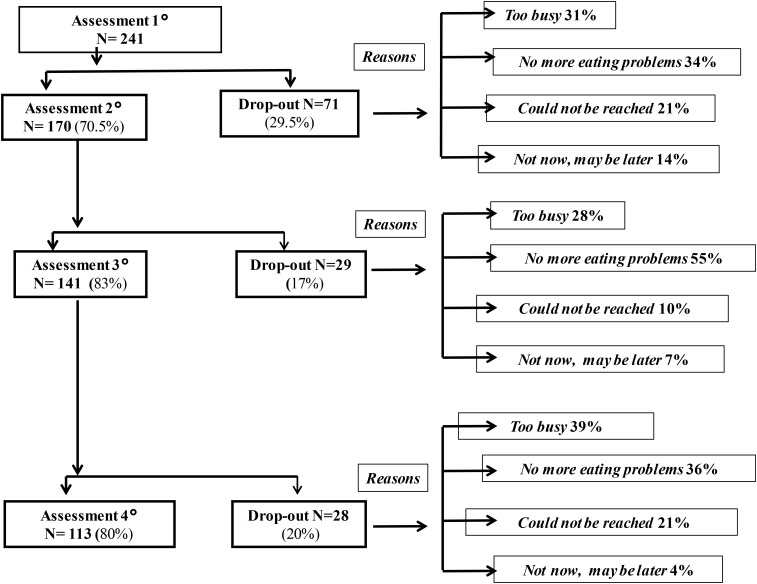
Patterns of drop-out. The denominator for each percentage is the number of children in the level immediately above. In total, 128 children (53%) dropped out from the Assessment 1° (Time of Diagnosis: at age 2) to Assessment 2° (at age 5), Assessment 3° (at age 7), and Assessment 4° (at age 11).

The participants were recruited at an Italian pediatric hospital in Rome. They were part of a larger sample of 241 children and their mothers, who had been diagnosed with IA, based on the DC: 0-3R’s criteria (Zero To Three, 2005). However, 128 participants were excluded from the data analyses of the present study because no longitudinal data were available from the Assessment 1° (at the time of the Diagnosis, children’s age: 2 years) to the subsequent Assessment 2° (children’s age: 5 years), 3° (children’s age: 7 years), and 4° (children’s age: 11 years). **Figure 1** shows the patterns of drop-out for the total sample. All four assessments were completed by 113 children and their mothers. **Table 1** reports gender, mean age and standard deviation of the 113 children who were examined four times (Assessment 1°, Assessment 2°, Assessment 3°, and Assessment 4°). All these children were full-term, without medical problems, and their psychomotor development was in the normal range; maternal mean age was 31.3 (SD = 4.6). Most of the children had been breastfed (86%) and were firstborn (79.5%). Most of the mothers were married (90%) and had obtained a secondary school diploma (88%). Most families were of middle socioeconomic status (SES) (95%).

**Table 1:** Means of age (SDs) and gender of children who were assessed at all four times.

Times of Assessments	Assessment 1°	Assessment 2°	Assessment 3°	Assessment 4°
Age of Children	Range = 1.6–2.9 years	Range = 4.3–5.5 years	Range = 7.2–8.1 years	Range = 10.8–11.5 years
	Mean = 2.3 years SD = 0.4	Mean = 5.1 years SD = 0.2	Mean = 7.9 years SD = 0.2	Mean = 11.2 years SD = 0.3
Males N = 58	Range = 1.6–2.8 years	Range = 4.7–5.4 years	Range = 7.2–8.1 years	Range = 10.8–11.5 years
	Mean = 2.3 years SD = 0.4	Mean = 5.1 years SD = 0.2	Mean = 7.9 years SD = 0.2	Mean = 11.2 years SD = 0.3
Females N = 55	Range = 1.6–2.9 years	Range = 4.3–5.5 years	Range = 7.2–8.1 years	Range = 10.8–11.5 years
	Mean = 2.3 years SD = 0.5	Mean = 5.1 years SD = 0.2	Mean = 7.9 years SD = 0.2	Mean = 11.2 years SD = 0.3


The diagnosis of IA was made by two independent clinicians (*k* = 0.93). Following the publication of DSM-5 (American Psychiatric Association [APA], 2013), the research team clarified that the young patients originally diagnosed with IA and enrolled in this study met the criteria for the ARFID subtype “apparent lack of interest in eating or food.”

The mothers of the children who were diagnosed with IA were re-contacted and invited to take part in a pediatric and psychological assessment at each time point. The study protocol was reviewed and approved by the IRB and all parents signed informed consent forms.

### Measures

#### Children’s Malnutrition

The children’s weight and height were recorded on the growth charts of the NCHS (Kuczmarski et al., 2000). Because the weight and height of most of the children with IA fell below the 3rd percentile, Waterlow et al.’s (1977) criteria were used to assess the degree of malnutrition. Weight for height reflects current or “acute” nutritional status. The reference “normal” is 50th percentile weight for height (Kuczmarski et al., 2000). The current weight divided by this number provides the percent of ideal body weight. Mild, moderate, and severe acute malnutrition correspond with 80–89%, 70–79%, and less than 70% of ideal body weight, respectively. Chronic malnutrition is assessed by the child’s height for age. The child’s actual height is divided by the height that corresponds to the 50th percentile for age, or “ideal height.” Mild, moderate, and severe chronic malnutrition correspond with 90–95%, 85–89%, and less than 85% of ideal height, respectively. The child’s degree of malnutrition (acute and chronic) was evaluated on a 4-point scale (from 0 = none, 1 = mild, 2 = moderate to 3 = severe).

#### Children’s Emotional/Behavioral Profile

Based upon the age of their child, mothers completed one of the two age versions of Achenbach and Rescorla’s (2000, 2001) Child Behavior Checklist (CBCL). These instruments fall into one of two age categories: 1½–5 years old and 6–18 years old. For each version, emotional/behavioral problems are evaluated. The internal coherence is satisfying and the validity of these tools is supported by their ability to differentiate accurately between *referred* and *non-referred* populations (Achenbach and Rescorla, 2000, 2001).

#### Mothers’ Psychopathological Profile

The Symptom Checklist-90-Revised (SCL-90R) (Derogatis, 1996), a 90-item self-report inventory, is a measure of current psychological symptom status scored on nine subscales and three global indices of distress [Global Severity Index (GSI), Positive Symptom Distress (PSID), and Positive Symptom Total (PST)]. Internal consistency is quite satisfactory (from 0.77 to 0.90) and high levels of construct and convergent-discriminant validity have been demonstrated (Derogatis, 1994).

#### Mothers’ Dysfunctional Eating Attitudes

The Eating-Attitudes Test (EAT-40) (Garner and Garfinkel, 1979) is a 40-item self-report inventory that identifies concerns with eating and weight in the adult population, scored on three subscales. A high score reflects dissatisfaction with body image, a desire to be thinner, preoccupation with eating and its effect on body size, and self-control when eating. It has shown a high degree of internal reliability (from 0.79 to 0.94) and has been validated on adult patients with Anorexia Nervosa (Garner and Garfinkel, 1979).

### Procedures

During each session, the children and their mothers underwent the following assessments: (1) a clinical screening was performed by a pediatrician who assessed the child’s growth and level of malnutrition and by a clinical psychologist who interviewed the mothers regarding the child’s patterns of eating behavior. The psychologist used a structured, unpublished interview, which was developed by Chatoor (2002) and Chatoor (1998, Unpublished) to diagnose children with different feeding disorders according to the classification by DC: 0–3R (2005); (2) the child’s psychological profile was evaluated by means of the CBCL 1½–5 or 6–18; (3) the mother’s psychological profile was assessed by administering the SCL-90R and the mothers’ dysfunctional eating attitudes were assessed by the EAT-40. The measures were presented in counterbalanced order.

### Data Analysis

The data were preliminarily screened for errors and outliers. Preliminary analysis showed that no variable had more than 5% of missing data. Missing data were adjusted according to each test norms or by introducing the scale average for the participant with missing data.

To examine whether children showed different types and degrees of malnutrition, a Kruskal–Wallis and Mann–Whitney *U*-tests were used. Friedman and Wilcoxon tests were performed to investigate changes in the level of malnutrition over time.

To examine the differences over time with respect to the degrees of malnutrition for each group of malnutrition (chronic, acute, acute/chronic), separate Friedman and Wilcoxon tests were conducted.

To examine differences between the CBCL scores over time, separate analyses were conducted considering the children’s age and the two different versions of the CBCL. Specifically, separate mixed ANOVAs were performed on the scores of the CBCL/1½–5 or CBCL/6–18, considering sex as an independent factor and Time as a repeated measure.

To examine the mother’s psychopathological profile and dysfunctional eating attitudes over time, a series of mixed ANOVAs were performed on the scores obtained by the mothers on the SCL-90R and EAT-40, using the sex of their children as independent variable and the four assessments as repeated measures.

Correlations (Bonferroni’s correction) were performed to investigate the relationships between the children’s severity of malnutrition, the children’s emotional/behavioral problems (CBCL), and maternal psychopathological symptoms (SCL-90R and EAT-40) at each assessment.

A hierarchical multiple regression was performed to discern if the children’s severity of malnutrition had a significant incremental impact on the children’s emotional/behavioral problems beyond the impact of maternal psychopathology and dysfunctional eating attitudes. At Step 1, the mothers’ anxiety and depression scores were entered. At Step 2, the mothers’ dysfunctional eating attitudes scores were entered, and at Step 3, the children’s severity of malnutrition was entered.

## Results

### Assessment of the Children’s Malnutrition

At the Assessment 1°, out of the sample of 113 children: 70.8% (50% boys, 50% girls) were suffering from “chronic,” 18.6% (62% boys, 38% girls) were suffering from “acute,” and 10.6% (42% boys, 58% girls) were suffering from “acute/chronic” malnutrition. **Table 2** shows their different degrees of malnutrition from the Assessment 1° to Assessment 4°.

**Table 2:** umber and percentages of children with severe, moderate, mild, and no malnutrition, at all four assessments.

	Assessment 1° *N*(%)	Assessment 2° *N*(%)	Assessment 3° *N*(%)	Assessment 4° *N*(%)
**Chronic (N = 80)**				
Severe	41 (51%)	41 (51%)	29 (36%)	20 (25%)
Moderate	20 (25%)	22 (28%)	26 (33%)	28 (35%)
Mild	19 (24%)	17 (21%)	25 (31%)	4 (5%)
No malnutrition	0 (0%)	0 (0%)	0 (80%)	28 (35%)
**Acute (N = 21)**				
Severe	16 (76%)	15 (72%)	13 (62%)	11 (52%)
Moderate	3 (14%)	3 (14%)	5 (24%)	4 (19%)
Mild	2 (10%)	3 (14)	3 (14%)	6 (29%)
No malnutrition	0 (0%)	0 (0%)	0 (0%)	0 (0%)
**Acute/Chronic (N = 12)**				
Severe	6 (50%)	6 (50%)	3 (25%)	3 (25%)
Moderate	4 (33%)	3 (25%)	3 (25%)	5 (42%)
Mild	2 (17%)	3 (25%)	6 (50%)	1 (8%)
No malnutrition	0 (0%)	0 (0%)	0 (0%)	3 (25%)


We found a significant change (χ^2^ = 6.64, *p* = 0.04) of the median scores in the degrees of malnutrition only at the Assessment 4° with respect to the different types of malnutrition (chronic, acute, acute/chronic). Specifically, a significant difference between the chronic group and the acute group (*z* = -2.57, *p* < 0.05), with the degree of malnutrition being greater for the acute group than for the chronic group. The degrees of malnutrition did not differ between the chronic and the acute/chronic groups (*p* = 0.18) or between the acute and the acute/chronic groups (*p* = 0.12).

In particular, a significant difference between the four assessments (χ^2^ = 107.80, *p* = 0.000) indicated that the degree of malnutrition was significantly higher at the Assessment 1° (Mdn = 2.82) than at either Assessment 3° (Mdn = 2.49) or Assessment 4° (Mdn = 1.86), *z* = -4.21, *p* < 0.001 and *z* = -6.31, *p* < 0.001, respectively. The severity of malnutrition was also significantly higher at Assessment 2° (Mdn = 2.81) than at Assessment 3°, *z* = -4.21, *p* < 0.001, and Assessment 4°, *z* = -4.7, *p* < 0.001. Overall, these results suggest a linear trend where the degree of malnutrition decreases significantly from the Assessment 1° to the Assessment 4°.

**FIGURE 2:**
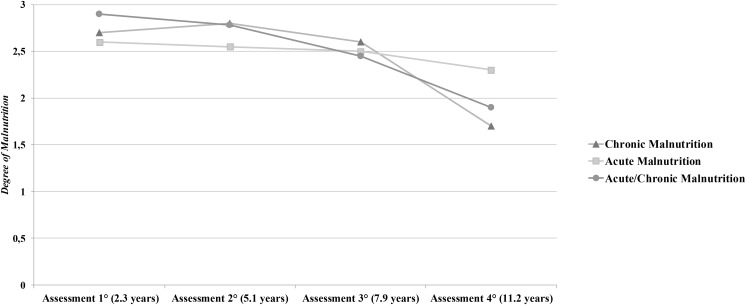
Assessments of the different types (chronic, acute, acute/chronic) and degree (mild, moderate, severe) of malnutrition. The vertical axis reports mean points of degree of malnutrition (from 0 = none to 3 = severe).

Finally, a significant difference over the four assessments for the chronic group (χ^2^ = 102.169, *p* = 0.000) and the acute/chronic group (χ^2^ = 8.350, *p* < 0.05) emerged. In the chronic group, the level of degree of malnutrition was significantly higher at the Assessment 1° (Mdn = 2.84) than at either Assessment 3° (Mdn = 2.52) or Assessment 4° (Mdn = 1.76), *z* = -3,50, *p* < 0.001 and *z* = -5.77, *p* < 0.001; in this group, the severity of malnutrition was significantly higher at Assessment 2° (Mdn = 2.88) than at Assessment 3° *z* = -3.88, *p* < 0.001 and Assessment 4° *z* = -5.87, *p* < 0.001. In the acute/chronic group, the level of degree of malnutrition was significantly higher at the Assessment 1° (Mdn = 2.92) than at Assessment 4° (Mdn = 1.96) *z* = -1.93, *p* < 0.05. No significant differences over time with respect to the degrees of malnutrition in the acute group emerged (**Figure 2**).

Although there was a steady improvement in the severity of malnutrition over time, it is important to note that 63% of children still have ongoing moderate to severe malnutrition at the Assessment 4° (**Table 2**).

### Assessment of Children’s Emotional/Behavioral Functioning

Analyses of the CBCL/1½–5 (**Table 3**) revealed a significant effect of Time on the Internalizing and Externalizing scales, where the children’s scores increased significantly at the Assessment 2°. No significant effects for Sex or its interactions with Time emerged.

**Table 3:** Means (SD) of the CBCL/1½–5 at the Assessment 1° and at Assessment 2°.

CBCL/1½–5	Assessment 1° (Mean = 2.3 years)	Assessment 2° (Mean = 5.1 years)	*F*_(1,111)_	η^2^	Cut-off
Somatic Complaints	7.08 (4.1)	8.4 (4.2)	8.66^∗∗^	0.07	> 6
Withdrawn/Depressed	6.6 (3.6)	7.2 (3.5)	2.92	0.01	> 4
Attention Problems	5 (2)	5.6 (2)	8.72^∗∗^	0.07	> 7
Aggressive Behavior	15.3 (6.9)	16.61 (6.8)	3.51	0.01	> 6
Anxious/Depressed	5.5 (3.3)	6.5 (3.1)	7.93^∗∗^	0.07	> 8
Emotionally Reactive	6.9 (4)	7.7 (3.8)	3.57	0.02	> 7
Internalizing Scale	26.1 (13.8)	29.8 (13.3)	6.66^∗^	0.05	> 16
Externalizing Scale	20.3 (8.1)	22.3 (8.1)	5.47^∗^	0.06	> 21


*The last column reports cut-off in the referring population. ^∗^p < 0.05; ^∗∗^p < 0.01.*

Analyses of the CBCL/6–18 (**Table 4**) revealed a significant effect of Time on the Internalizing and Externalizing Scales, where the children’s scores increased significantly at the Assessment 4°.

**Table 4:** Means (SD) of the CBCL/6-18 by gender at Assessment 3° and at Assessment 4°.

CBCL/6-18	Assessment 3°		Assessment 4°		*F*	Cut-off	
	*Males* (Mean = 7.9 years)	*Females* (Mean = 7.9 years)	*Males* (Mean = 11.2 years)	*Females* (Mean = 7.9 years)		*Males*	*Females*
Anxious/Depressed	11.1(4.9)^a^	11.2 (5.2)^a^	12.6 (5)^a^	14.4(6.3)^b^	12.02^∗∗^	> 12	> 10
Withdrawn/Depressed	6.7(6.7)^a^	6.2 (3.1)^a^	7.6 (3.7)^a^	8.5(4.3)^b^	10.38^∗∗^	> 6	> 6
Somatic Complaints	3.3(3.3)^a^	3.7 (2.4)^b^	4.2(2.7)^c^	6.8(2.8)^d^	3.75^∗∗^	> 4	> 5
Social Problems	9.7(4.5)^a^	9.5(4.6)^a^	11.3(4.2)^b^	12.5(5.4)^b^	15.52^∗∗^	> 6	> 6
Thought Problems	7.3(6)^a^	8.5(6.5)^b^	7.5(6.1)^c^	9.4(7.4)^d^	8.99^∗∗^	> 2	> 2
Attention Problems	8.8 (4)^a^	9.4 (4.5)^b^	9.8(7)^c^	11.4(4.5)^d^	7.04^∗∗^	> 11	> 10
Rule-Breaking Behavior	12.6(6.3)^a^	12.9(6.7)^a^	15.4(7.3)^b^	17.2(8.8)^b^	13.29^∗∗^	> 4	> 4
Aggressive Behavior	14.8 (7)^a^	15.4(7.1)^b^	16.6(7)^c^	19.3(8.1)^d^	7.04^∗∗^	> 20	> 17
Internalizing Scale	21(9.5)^a^	21(9.8)^a^	24.4 (10.1)^a^	28.6(12.7)^b^	12.19^∗∗^	> 11	> 12
Externalizing Scale	27.4(12.8)^a^	28.3(13.2)^a^	31.9 (13.4)^a^	37.2(16.4)^b^	10.68^∗∗^	> 17	> 13


*Means in rows not sharing a common letter differ significantly (p < 0.05). The last column reports cut-off scores in the referring population. ^∗^p < 0.05; ^∗∗^p < .01.*

Analyses showed a significant effect of Sex on: *Somatic Complaints* [*F*_(1,111)_ = 18.04, *p* < 0.001, η^2^ = 0.08], *Thought Problems* [*F*_(1,111)_ = 3.5, *p* < 0.05, η^2^ = 0.03], *Attention Problems* [*F*_(1,111)_ = 3.83, *p* < 0.05, η^2^ = 0.04], and *Aggressive Behavior* [*F*_(1,111)_ = 3.05, *p* < 0.05, η^2^ = 0.03], where the girls had higher scores than the boys. No significant interactions between Sex and Time emerged.

### Assessment of Mothers’ Psychopathological Profile and Dysfunctional Eating Attitudes

Analyses of the SCL-90R showed a significant effect of Time in the GSI [*F*_(2.2,242)_ = 14.140, *p* < 0.001, η^2^ = 0.11], in the PST [*F*_(2.6,284.25)_ = 6.26, *p* < 0.001, η^2^ = 0.05], and in the PSID [*F*_(2.7,301.4)_ = 8.5, *p* < 0.001, η^2^ = 0.07], where the mothers showed a significant increment at the Assessment 4° (*p* < 0.01). Also, analyses showed a significant effect of Time on: *Somatization* [*F*_(2.6,288.38)_ = 5.18, *p* < 0.01, η^2^ = 0.11], *Interpersonal Sensitivity* [*F*_(2.5,282)_ = 12.55, *p* < 0.001, η^2^ = 0.10], *Obsessive-compulsive* [*F*_(2.6,383.05)_ = 8.14, *p* < 0.001, η^2^ = 0.07], *Depression* [*F*_(2.5,280.8)_ = 17.4, *p* < 0.001, η^2^ = 0.13], *Anxiety* [*F*_(2.7,303.2)_ = 8.63, *p* < 0.001, η^2^ = 0.07], *Hostility* [*F*_(2.6,290.4)_ = 9.57, *p* < 0.05, η^2^ = 0.03], *Paranoid Ideation* [*F*_(2.7,294.9)_ = 9.28, *p* < 0.001, η^2^ = 0.08], *Psychoticism* [*F*_(2.78,308.4)_ = 7.45, *p* < 0.001, η^2^ = 0.06], and *Phobic Anxiety* [*F*_(2.69,298.5)_ = 5.8, *p* < 0.001, η^2^ = 0.05], where the mothers’ scores increased significantly at the Assessment 4° (*p* < 0.01). Furthermore, analyses showed a significant effect of the child’s Sex × Time interaction on *Hostility* [*F*_(2.6,290.4)_ = 3.01, *p* < 0.05, η^2^ = 0.03], where the girls’ mothers had higher scores than the boys’ mothers. Specifically, the mothers of the girls showed significantly higher scores than the mothers of the boys at the Assessment 4° (*p* < 0.01).

Analyses of the EAT-40 showed a significant effect of Time [*F*_(2.6,284.7)_ = 3.89, *p* < 0.05, η^2^ = 0.03], where the mothers’ scores increased significantly at the Assessment 4° (*p* < 0.01). Analyses also revealed a significant effect of the child’s sex [*F*_(2.6,290.4)_ = 3.01, *p* < 0.05, η^2^ = 0.03], showing that the girls’ mothers had significantly higher scores than the boys’ mothers. Moreover, analyses showed a significant effect of Time on *Bulimia/Food Preoccupation* [*F*_(2.52,274.3)_ = 4.05, *p* < 0.01, η^2^ = 0.04] and *Oral Control* [*F*_(2.7,300.01)_ = 5.25, *p* < 0.01, η^2^ = 0.05], where the mothers’ scores increased significantly at the Assessment 4° (*p* < 0.05). Finally, analyses showed a significant effect of the child’s Sex × Time interaction on *Dieting* [*F*_(2.6,290.3)_ = 4.97, *p* < 0.01, η^2^ = 0.02], where the girls’ mothers had significantly higher scores than the boys’ mothers. Specifically, the mothers of the girls showed higher scores than the mothers of the boys on *Dieting* at the Assessment 4° (*p* < 0.01) (**Table 5**).

**Table 5:** Means (SD) of the SCL-90 and of the EAT-40 scores by group and time of assessment.

	Assessment 1°	Assessment 2°	Assessment 3°	Assessment 4°	Cut-off
**SCL-90-R**					
GSI	1.8 (1.1)^a^	1.2 (0.6)^b^	1.2 (0.6)^b^	1.5 (0.6)^c^	> 0.78
PSDI	1.7 (0.4)^a^	1.8 (0.4)^a^	1.7 (0.4)^a^	1.9 (0.5)^b^	> 1.88
PST	55.3 (2.1)^a^	57.5 (20.9)^a^	57.9 (19.1)^a^	65.8 (19.2)^b^	> 46
Somatization	1.3 (0.8)^a^	1.4 (0.7)^a^	1.3 (0.7)^a^	1.7 (0.8)^b^	> 1.03
Obsessive compulsive	1.2 (0.8)^a^	1.3 (0.7)^a^	1.1 (0.5)^a^	1.6 (0.8)^b^	> 1.03
Interpersonal sensitivity	1.1 (0.6)^a^	1.2 (0.6)^a^	1.1 (0.7)^a^	1.5 (0.7)^b^	> 0.91
Depression	1.5 (0.5)^a^	1.5 (0.5)^a^	1.3 (0.6)^b^	1.8 (0.6)^c^	> 1.11
anxiety	1.2 (0.8)^a^	1.3 (0.8)^a^	1.3 (0.7)^a^	1.7 (0.8)^b^	> 0.91
Hostility	0.9 (0.7)^a^	1.0 (0.7)^a^	1.1 (0.7)^a^	1.4 (0.8)^b^	> 0.83
Phobic anxiety	1.1 (0.8)^a^	1.2 (0.8)^a^	1.2 (0.7)^a^	1.5 (0.9)^b^	> 0.58
Paranoid ideation	1.1 (0.7)^a^	1.1 (0.7)^a^	1.4 (0.9)^a^	1.6 (0.8)^b^	> 0.91
Psychoticism	1.7 (0.8)^a^	1.1 (0.8)^a^	1.2 (0.4)^a^	1.5 (0.8)^b^	> 0.42
**EAT-40**					
TOTAL	51.1 (20.3)^a^	54.1 (19.8)^a^	53.4 (22.4)^a^		> 29
Oral control	9.2 (3.4)^a^	10.1 (3.7)^a^	8.9 (4.5)^a^		
Bulimia and food Preoccupation	7.3 (4.1)^a^	7.4 (4)^a^	6.8 (4.0)^a^		
Dieting	17.2 (8.6)^a^	17.8 (7.8)^a^	17.6 (7)^a^		


*Means in rows not sharing a common letter differ significantly (p < 0.05). The last column report the cut-off scores in the referring population when available.*

### Correlations Among Variables

Significant correlations between maternal psychopathological symptoms and the emotional/behavioral problems of their children emerged at all assessments. Also, significant correlations between the children’s severity of malnutrition and emotional/behavioral problems emerged at the Assessment 4° (**Table 6**).

**Table 6:** Correlations between the severity of malnutrition, child’s emotional–behavioral symptoms and mothers’ psychopathological symptoms, and dysfunctional eating attitudes at each assessment.

	Assessment 1°					Assessment 2°					Assessment 3°					Assessment 4°				
	1	2	3	4	5	1	2	3	4	5	1	2	3	4	5	1	2	3	4	5
1.SEV-MALN.	–					–					–					–				
2.CBCL-TOT.	-0.20	–				0.11	–				0.12	–				0.37^∗∗^	–			
3.SCL-DEP.	0.03	0.39^∗∗^	–			0.11	0.27^∗^	–			0.13	0.55^∗∗^	–			0.23^∗^	0.58^∗∗^	–		
4.SCL-ANX	0.1	0.40^∗∗^	0.74^∗∗^	–		0.01	0.17	0.66^∗∗^	–		0.07	0.65^∗∗^	0.83^∗∗^	–		0.19	0.62^∗∗^	0.69^∗∗^	–	
5. EAT-TOT	0.0	0.2	0.34^∗∗^	0.47^∗∗^	–	0.26^∗^	0.07	0.36^∗∗^	0.52^∗∗^	–	0.02	0.57^∗∗^	0.76^∗∗^	0.76^∗∗^	–	0.18	0.47^∗∗^	0.37^∗∗^	0.45^∗∗^	–


*SEV-MALN: severity of malnutrition; CBCL-TOT: CBCL total score; SCL-DEP: SCL-90-Mothers’ depression; SCL-ANX: SCL-90-Mothers’ anxiety; EAT-TOT: EAT-40 total score. ^∗^p < 0.05; ^∗∗^p < 0.01 after Bonferroni’s correction for number of comparisons.*

### Multivariate Prediction of Children’s Emotional/Behavioral Problems

As illustrated in **Table 7**, maternal psychopathology significantly predicts children’s emotional/behavioral problems, explaining a large amount of variance (42%). Higher maternal anxiety and depression scores are associated with higher children’s emotional/behavioral problems. Moreover, maternal dysfunctional eating attitudes explain an additional 4% of the variance; higher scores indicate greater children’s emotional/behavioral problems. Finally, the children’s severity of malnutrition explains an additional 5% of the variance. The proportion of variance accounted for by the full model was 51%, *F*_(4,80)_ = 20.02; *p* < 0.001.

**Table 7:** Hierarchical multiple regression models predicting emotional problems in children with Infantile Anorexia or ARFID subtype 1.

	Emotional Problems Assessment 4°		
	Step-1 β	Step-2 β	Step-3 β
SCL-90-mothers’ depression at Assessment 4°	0.29^∗^	0.26^∗^	0.23^∗^
SCL-90-mothers’ anxiety at Assessment 4°	0.42^∗∗^	0.33^∗∗^	0.33^∗∗^
EAT-40 total score at Assessment 4°		0.23^∗^	0.20^∗^
Severity of malnutrition at Assessment 4°			0.23^∗∗^
*R*^2^	0.42^∗∗^	0.46^∗∗^	0.51^∗∗^
Adjusted *R*^2^	0.41^∗∗^	0.44^∗∗^	0.49^∗∗^
*ΔR^2^*		0.04^∗∗^	0.05^∗∗^


*^∗^p < 0.05; ^∗∗^p < 0.01.*

## Discussion

Our study describes the longitudinal course of IA, defined by the DC: 0-3R criteria, which correspond to the ARFID subtype “lack of interest in food or eating” diagnosis in DSM-5. Particularly, this study reports the developmental course of IA overtime, in terms of the clinical course of malnutrition type and severity level, child emotional functioning, and maternal psychopathology.

We found that children with this eating disorder showed various degrees of malnutrition, ranging from mild to moderate to severe malnutrition; specifically, 90 children (80%) suffered from severe or moderate malnutrition at the time of diagnosis. A steady improvement in the severity of malnutrition over time emerged, however, at 11 years of age, 71 children (63%) still showed moderate to severe malnutrition, 11 children (10%) were mildly malnourished, and 31 children (27%) were no longer malnourished. However, although the malnutrition improved over time, the children’s internalizing and externalizing problems became more evident as they grew older, showing that these children are at risk not only for ongoing eating difficulties, but also for emotional/behavioral problems.

Moreover, we observed that the majority of mothers suffered from clinically significant eating difficulties and psychopathology, mainly anxiety, and depression. While the children showed increasing internalizing and externalizing symptoms, particularly at the last assessment, the mothers’ psychopathological symptoms and eating problems also worsened over time. The striking correlations between the children’s and mothers’ symptoms shed light on an ongoing circular pattern of disturbed eating behavior and emotional symptoms in both the children and their mothers. This extends the findings from previous cross-sectional research during infancy and some longitudinal research from infancy to middle childhood (Marchi and Cohen, 1990; Kotler et al., 2001; Cooper et al., 2004; Stein et al., 2006; Nicholls and Viner, 2009; Bryant-Waugh et al., 2010; Ammaniti et al., 2012; Chatoor, 2013; Bryant-Waugh and Watkins, 2015).

In this longitudinal study, in particular when looking at the last assessment, when the children were 11 years old, i.e., close to puberty, a significant effect of the sex of the children emerged for *Somatic Complaints*, *Thought Problems*, *Attention Problems*, and *Aggressive Behavior* on the CBCL. The girls had average ratings significantly more severe than the boys. It is important to note that *Aggressive Behavior* and *Attention Problems*, which were more severe in the girls than in the boys, relate to oppositional behaviors and highlight difficulties in regulating affects between these children and their mothers during this sensitive developmental age close to puberty. Looking at the mothers’ psychopathological and eating symptoms, when their children were 11 years old, the girls’ mothers showed significantly higher scores on *Hostility* and *Dysfunctional Eating Attitudes*, specifically on *Dieting* than the boys’ mothers. Overall, these risk variables that emerged in both the girls and their mothers are concerning, considering the developmental age of these girls, i.e., close to puberty, which has been identified as a critical risk period for the emergence of eating disorders (Combs et al., 2011; Klump et al., 2013).

Interestingly, when we explored the predictor risk factors of the emotional/behavioral problems in the group of children who suffered from persistent malnutrition up to 11 years of age, we found that maternal psychopathological symptoms of anxiety, depression, and dysfunctional eating attitudes are the best predictor of the child’s emotional/behavioral problems, followed by the child’s severity of malnutrition, which had a significant incremental impact on the children’s emotional/behavioral problems. These findings further highlight the importance of evaluating maternal psychopathological symptoms which may contribute to the onset, severity, and persistence over time of this specific infantile eating disorder. In addition, the study shows that the severity of the child’s malnutrition plays a significant role in the development of emotional and behavioral symptoms of the child. The lack of energy may predispose these children to poor functioning at home and at school.

## Conclusion

Our prospective study from infancy to pre-puberty revealed biologic, emotional-behavioral, and familial risk factors in a sample of children with a history of IA and their mothers, who received limited psychosocial treatment. Considering our results, which demonstrated a significant effect of sex – namely that at the age of 11 years, especially the girls and their mothers demonstrated increased eating pathology and associated psychopathological symptoms – further longitudinal research is necessary to better understand how these children negotiate adolescence. It raises the question whether these children will develop a fear of becoming fat and transition to the full picture of Anorexia Nervosa or other restrictive eating disorders, during the critical developmental period of adolescence, which represents a peak period for the onset of eating disorders, especially in girls (Lewinsohn et al., 2000; Currin et al., 2005; Van Son et al., 2006; Keski-Rahkonen et al., 2007).

Puberty and early adolescence (11–14 years of life) represent transitional phases of life characterized by physical, psychological, and social changes (Steinberg, 1999). During this period, teenagers experience major body changes and going through puberty may serve as major stressor for triggering or worsening body dissatisfaction and low self-esteem. The brain and the cognitive functions mature, and there is an increased awareness of societal pressures for thinness and an increased concern about peer acceptance (Bryant-Waugh and Lask, 2007). Moreover, a number of major psychological transitions occur throughout adolescence, including the formation of identity, increasing independence from parents, and the initiating of romantic relationships that may work as stressors to trigger an eating disorder (Call et al., 2017). During this critical developmental period, the identification with parents, especially the relationship of adolescent girls with their mothers, plays an important role in promoting or hindering the processes of separation, individuation, and autonomy of adolescent girls (Barenson et al., 2005). Based on this theoretical research background and in light of the findings of our study, the greater difficulties that emerged in regulating affects between female children and their mothers, especially at 11 years of age, are of concern. Moreover, regarding body image, it is important to consider that the mother is the first role model for her daughter. In our sample, the mothers’ dietary symptoms and body dissatisfaction raise concern about the influence of these mothers’ role model on their daughters (Cooley et al., 2008). In future research, it will be important to measure the effects of malnutrition on the onset of puberty and the effects of mothers’ eating concerns on the children’s body image when they mature sexually.

A limitation of this study is the drop-out of more than half of the mothers and their children of the original sample, which is not uncommon for longitudinal studies. As we show in **Figure 1**, some of these children seem to do well according to the parents, whereas the parents of the other children were too busy, or could not be reached. Consequently, the results of our study cannot be fully generalized. However, the findings of this longitudinal study speak to the seriousness of this infantile feeding disorder and the need for early diagnosis and treatment within the family context.

Another limitation of this longitudinal study is that the children have not reached adolescence, but our findings shed light on the chronology of mothers’ mental health and their children’s development from feeding disorders in early childhood to eating disorders and emotional and behavioral difficulties in later childhood up to the sensitive period of pre-puberty. It further confirms the importance of a comprehensive approach to the assessment and treatment of early feeding disorders which not only covers the child’s eating difficulties, but also addresses the child’s emotional development within the family context (Sameroff, 2004; Lucarelli et al., 2017). Future expansion of our longitudinal study into adolescence, based on further evaluations of this sample, will be pivotal to fully understand the boys’ and girls’ outcomes in both eating behavior and emotional development.

Finally, another limitation of this prospective study is the lack of a systematic investigation of the role of fathers, an area which has not yet been sufficiently explored and needs to be clarified in future research.

## Ethics Statement

The present study has been realized complying with the APA and the Italian Association of Psychology (AIP) ethical principles and it was approved by the involved Hospital and University Ethics Committees. All the parents signed the written informed consent to participate with their children at the research program.

## Author Contributions

LL contributed to prepare the study design, to the recruitment of the sample, and to write all the sections of the manuscript. CS contributed to prepare the study design, performed the statistical analyses, prepared tables and figures, and contributed to write the “Materials and Methods” and “Results” sections. SC contributed to prepare the study design, to the recruitment of the sample, and to the data collection. IC contributed to prepare the study design and to write the “Introduction” and “Discussion” sections.

## Conflict of Interest Statement

The authors declare that the research was conducted in the absence of any commercial or financial relationships that could be construed as a potential conflict of interest.
